# Modifier Genes and the Plasticity of Genetic Networks in Mice

**DOI:** 10.1371/journal.pgen.1002644

**Published:** 2012-04-12

**Authors:** Bruce A. Hamilton, Benjamin D. Yu

**Affiliations:** 1 Department of Cellular and Molecular Medicine, University of California San Diego, La Jolla, California, United States of America; 2 Department of Medicine, Moores UCSD Cancer Center and Institute for Genomic Medicine, University of California San Diego, La Jolla, California, United States of America; University College London, United Kingdom

## Abstract

Modifier genes are an integral part of the genetic landscape in both humans and experimental organisms, but have been less well explored in mammals than other systems. A growing number of modifier genes in mouse models of disease nonetheless illustrate the potential for novel findings, while new technical advances promise many more to come. Modifier genes in mouse models include induced mutations and spontaneous or wild-derived variations captured in inbred strains. Identification of modifiers among wild-derived variants in particular should detect disease modifiers that have been shaped by selection and might therefore be compatible with high fitness and function. Here we review selected examples and argue that modifier genes derived from natural variation may provide a bias for nodes in genetic networks that have greater intrinsic plasticity and whose therapeutic manipulation may therefore be more resilient to side effects than conventional targets.

## A Brief Conceptual History of Modifier Genes

The concept of modifier genes originated with the solution to an early genetic mystery. As early workers tested Mendel's segregation ratios with observations on a wide range of traits and species, several cases of “inconstant inheritance” caused some to question the Mendelian basis of factors underlying these traits [1], [2]. Three cases of particular note are *Beaded* and *truncate* wings in fruit flies and pigmentation in hooded rats. In each of these cases, the phenotype varied widely among offspring of parents with established genotypes. Mutant frequencies did not follow Mendelian ratios and—more troubling still—varied among derived lines carrying the same mutation. This led some workers to question whether genes were constant physical units or changed in properties during transmission [3], [4] and was the last serious challenge to the theory of the gene and the generality of Mendelian inheritance. Resolution of this issue came with demonstrations that defined genetic backgrounds could account for the variation in *truncate* flies [5] and hooded rats [6], [7] and more precisely, by linkage mapping, that discrete genetic loci account for the observed variation in *Beaded* flies [8]. The term “modifier gene” was coined to indicate a genetic variant identified by its impact on a conditioning mutation, with no obvious phenotype of its own. This is the usage we will follow here (Box 1).

**Box 1. What Is a Modifier Gene?**

The term has been applied to several classes of genetic activities, some of which overlap. Genetic variations that alter the activity of a protein encoded by a second locus are one class. To the extent that the modifying effect is independent of allelic variation in the gene being modified, this usage is conceptually equivalent to any primary mutation whose phenotypic consequences include loss of interaction with its normal targets (as for a transcription factor and its target genes, or a protein kinase and its substrates). Interactions between mutations that were each previously recognized by their independent phenotypes are another class. While these genetic interactions can be revelatory, one need not invoke the term “modifier” to fully describe both individual and interaction effects, as each locus is identifiable without the other. Quantitative trait loci (QTL) are sometimes referred to as modifiers in the context of a major effect locus, which can be nearer the original meaning, depending on the structure of interactions in the QTL model. For the purposes of this review, we will use “modifier” to mean a genetic variant that is best recognized by its ability to change the phenotypic outcome of an independent “conditioning” variant at another locus. Modifier genes in this usage have no obvious phenotype of their own prior to discovery and are effectively silent—or at least quiet—with respect to the phenotype under study, in the absence of the conditioning mutation.

Early workers quickly appreciated that modifier genes are pervasive across experimental systems and organisms. Modifier genes need not be subtle and can have phenotypic effects as large as the initial conditioning mutation. These early observations contributed to the conceptualization of genetic pathways, prior to knowing the molecular identities of any components. This formal concept remains useful in analyzing genetic networks for sensitive nodes (genes) through which to manipulate mutant phenotypes in genetic disease or experimental biology.

## Modifier Genes in Human Disease and Mouse Models: Ripe for Harvest

Modifier genes are also frequent in human disease and often invoked to explain divergent outcomes in genetic disorders with apparently equivalent cause. Among the best examples is cystic fibrosis (CF). CF patients homozygous for the Δ508 allele (approximately half of patients with European ancestry) present with a broad range of organ involvement and clinical severity, much of which is controlled by modifier genes [9]. Despite being among the most common Mendelian disorders and having an unusually common disease allele to minimize heterogeneity, molecularly defined modifiers have only very recently been reported [10], [11]. Just as CF was one of the first major victories in positional cloning of disease genes, its modifier gene network is one of the first to see real progress apart from candidate genes and serendipitous studies. Comparison between modifier studies in human patients and mouse models of CF [12]–[15] will be especially interesting, given replication of at least one syntenic locus between species [16]. Among rarer disorders, some ciliopathy phenotypes (and related developmental abnormalities) may require or be modified by tri-allelic inheritance—requiring three alleles among two or more loci—among functionally related genes [17]–[21]. However, these examples are the exceptions. Most modifier effects in human genetic disorders are much less well understood, and the numbers of available subjects, allelic heterogeneity, and environmental factors that obscure modifier effects in smaller cohorts may limit systematic analyses for a large number of individually rare disorders. This increases the potential value of animal models for both candidate discovery and experimental validation of human modifier genes.

Identification of modifier genes in mouse models offers an opportunity to understand forms of plasticity in mammalian genetic architectures that could be exploited as a preclinical knowledge base in designing therapeutic strategies [22]. A very common finding in mouse models is phenotypic difference between strain backgrounds (e.g., 129 versus C57BL/6 in hybrid lines from gene-targeting experiments), but the sources of variation are not often pursued because of technical and resource constraints. Limited access to these modifier genes is a missed opportunity. Since modifiers are necessarily context dependent, any experimental organism is likely to model only a portion of any particular genetic network in disease. The accuracy of multigenic models likely depends on both the evolutionary plasticity of the network involved and the evolutionary distance between the model and human subjects. From this perspective, with rodents as a sister group to primates, mouse variants that have large effect sizes as modifiers while remaining phenotypically quiet on their own should be useful in identifying points in a genetic network where intervention might have higher therapeutic benefit than collateral cost.

**Figure 1:**
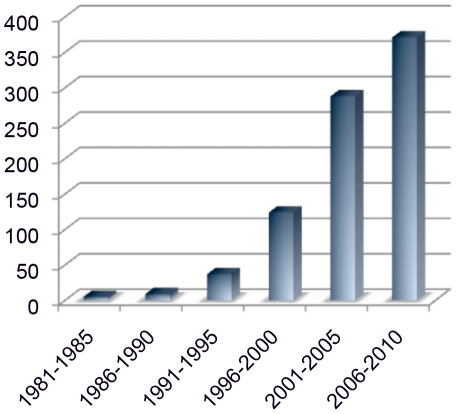
PubMed references to mouse modifier genes. Growing interest in and recognition of modifier genes in mouse models is supported by increased publication rates in 5-year windows for the last 30 years. Mouse modifier papers have increased at a rate faster than the increase of total PubMed citations over the past 15 years.

The examples below argue that modifier genes are a field ripe for harvest, particularly with the recent arrival of several new community resources that improve experimental access to genetic variations among common strains. The situation is reminiscent of early days of positional cloning in many respects. Large numbers of loci have been reported and mapped (Figure 1; Table S1), but few have been molecularly identified. Stories of unrewarded efforts have inhibited some investigators from pursuing otherwise tantalizing genetic effects. The two most important parallels, however, are that early successes—highlighting unexpected interactions—show the value of the approach and that new resources and emerging technologies are making the approach both more palatable and more cost effective. With new tools and a critical mass of encouraging results from several laboratories, this is a good time to consider both the value of the waiting crop and the machinery needed for harvest. Instructive examples to date include identification of genetic interactions from candidate genes, from mutagenesis screens, and from natural variants. Understanding modifier gene network architectures in mouse models may provide a powerful functional basis for inferring candidate interactions from human exome and genome sequences, particularly where sample size limits statistical power for independent discovery in clinical samples for Mendelian disorders.

## Candidate Genes Are a Useful, but Limited, View of Interactions

Many of the genetic interactions known in mice come from direct tests of candidate interactions. Testing interactions first observed in other species can identify physiological context for gene pairs or networks that are conserved more deeply than the physiology or organ system for which they are most relevant to human disease. For observed binding partners, genetic interactions can test the biological relevance of likely physical contacts. Proposed interactions between mutations with similar phenotypes can also clarify points of convergence between previously separate pathways.

Candidate interactions are often based on homology to interacting genes in other species, including components of developmental signaling pathways, homeotic regulators, and transcription factor cascades in development. Some of these interactions may confirm modifier genes in the strict sense of Box 1, but more often involve interactions between phenotypic null mutations available from other studies. While these are helpful in confirming functional conservation among pathways and defining mammalian contexts that might be relevant to disease, this paradigm is limited in its ability to identify levels of interaction unique to mammalian biology that might be expected from successive genome duplications, rescissions, and neo-functionalization in the lineage leading from ancestral vertebrates to primates and rodents (Euarchontoglires). Indeed, levels of redundancy among paralogous genes in mouse experiments often underscore important differences in trait architectures between mammals and other models. Moreover, focus on just the highly conserved rather than more plastic components of a genetic pathway or network might bias against finding genes that are more readily manipulated without damage to the organism.

Synthetic interactions between environmentally sensitizing alleles are another kind of candidate gene approach. For example, BALB/c and 129S1 mice are sensitive to adriamycin-induced nephropathy and show mitochondrial DNA (mtDNA) depletion in vulnerable organs after adriamycin treatment. Gharavi and colleagues identified a single amino acid substitution in the shared BALB/c and 129S1 allele of *Prkdc*, which encodes a DNA-activated protein kinase, as the sensitizing variant and asked whether this might be relevant to other mtDNA dysfunctions [23]. The human orthologue of *Mpv17*, which encodes an inner mitochondrial membrane protein, is mutated in mtDNA depletion syndromes [24], but the corresponding mouse mutation does not produce this phenotype. Papeta et al. showed that *Prkdc* potentiates mtDNA loss in *Mpv17*-mutant mice: double mutant mice (but neither single mutant) suffer mtDNA loss and other features of adriamycin-induced nephropathy without adriamycin exposure, providing parallel gene×gene and gene×environment models.

The many successes in modeling the phenotypic consequences of predicted candidate gene interactions in an intact mammal should encourage us to consider what value might be obtained from screens that are less constrained by prior predictions—and therefore capable of identifying novel and unexpected interactions that might catalyze more rapid progress in the often complex genetic architectures relevant to disease.

## Spontaneous Modifiers: Volunteers Lead the Way

An early success in using modifier genes to understand a genetic network in mice came from the *dilute suppressor* (*Mreg^dsu^*). This modifier arose spontaneously in a *non-agouti*, *dilute* stock, suppressing the coat color dilution but with no obvious phenotype of its own [25]. Importantly, *dsu* similarly suppressed pigmentation phenotypes in mutations at five of 11 classical coat color loci tested [26], [27], indicating a fundamental role in melanosome function. Molecular analysis revealed a spontaneous 11-kb deletion, creating a null allele in a vertebrate-specific gene, subsequently dubbed *Melanoregulin* [28]. Characterization of Melanoregulin in the context of its genetic suppressor activity revealed a previously unknown molecular function required for pigment granule transfer and other transactions among membrane-bound organelles [29]. This work remains an instructive example as few other strict-sense modifiers have been demonstrated to act on several different mutations and only one mouse modifier, *Nxf1^Mvb1^*, has been effective on a larger number.

Spontaneous mutations can also contribute to nominally wild-type inbred backgrounds. The *Sodium channel modifier 1* (*Scnm1*) locus was identified as a strain-dependent modifier of *Scn8a^med-J^*, a mutation in a neuronal sodium channel gene responsible for a range of neurological phenotypes [30]. Among several *Scn8a* mutations, the modifier is specific for the *med^J^* allele, an intronic single nucleotide variant that alters 5′ splice site usage. Positional cloning of the modifier identified a novel gene whose protein plays a direct and previously unsuspected role in pre-mRNA splicing [31], [32]. The sensitizing modifier allele (C57BL/6) encodes a truncated protein (R187X), but is less severe than a subsequently generated null allele [33]. The interaction between *Scnm1* and *Scn8a^med-J^* appears highly specific, as *Scnm1* does not alter general RNA patterns in brain nor modify other tested mutations with similar defects [33]. Because the variant *Scnm1* allele is only found in C57 and C58 strains, but not in other strains with otherwise similar *Scnm1* haplotypes, it is proposed to have arisen as a spontaneous mutation in a progenitor stock rather than as a wild population variant [32]. The unexpected aspects of mammalian biology identified through the *dsu* and *Scnm1* spontaneous modifiers should encourage further explorations of modifier genes.

## Induced Mutations Allow Systematic Screens—with Dramatic Effects

Random mutagenesis to introduce and screen new variants has several advantages for identifying genetic interactions and trait architectures. Ethylnitrosourea (ENU) is especially efficient in mice [34], [35] and in principle can both produce a range of alleles at a given locus and saturate a phenotype for simple loss-of-function alleles at most loci. High-throughput sequencing now rapidly identifies de novo mutations relative to a defined background [36]–[38]. With respect to modifier screens, mutagenesis should in principle identify both strict-sense modifiers and interacting genes with significant independent phenotypes, though the relative proportion may be difficult to predict.

**Figure 2:**
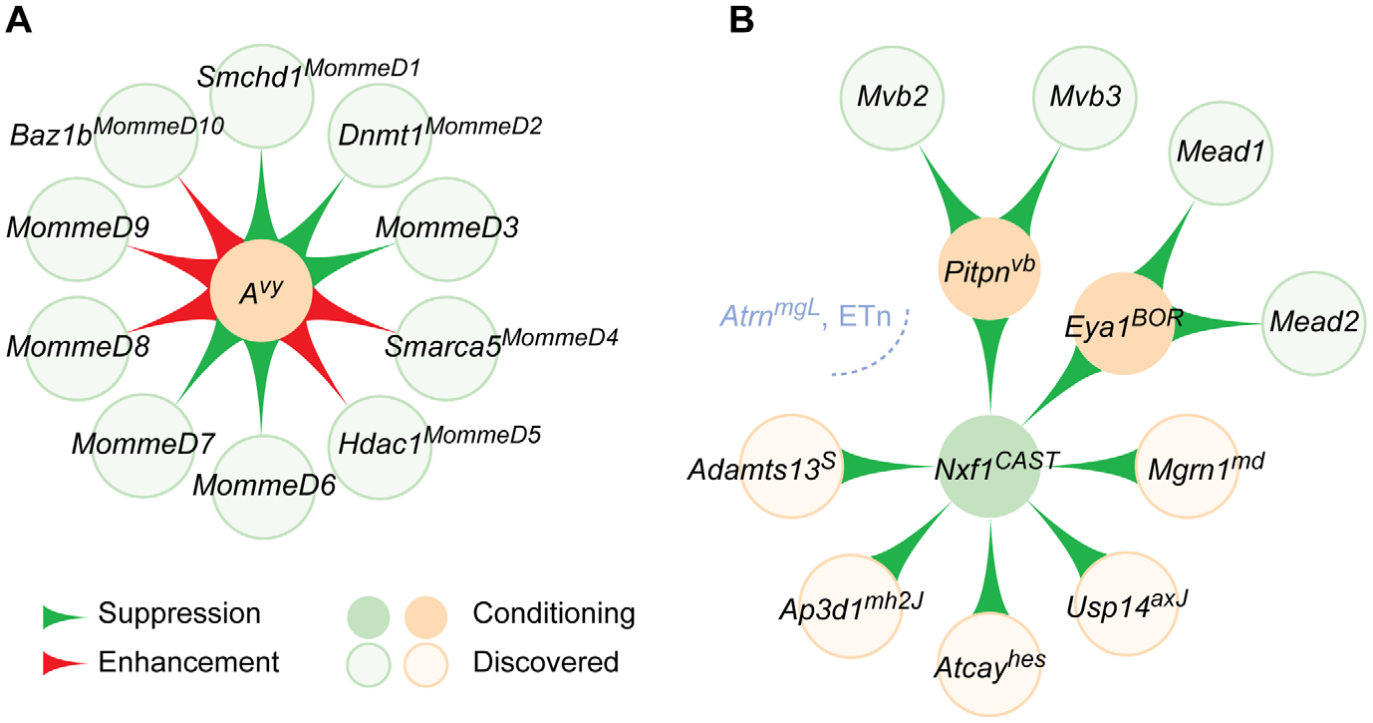
Modifier gene networks have directional edges between nodes. Mouse modifier gene interactions can be diagrammed as nascent modules of an interaction network. (A) Identification of multiple modifier genes for a conditioning mutation through either mutagenesis or linkage analysis of strain variants can be represented as in “outside-in” network module, where one node (the conditioning mutation, beige circle) is a sink hub, acted on by each experimentally discovered modifier (light green circles). *MommeD* modifiers of the epigenetically sensitive *A^vy^* mutation are illustrated as an example. Direction of effect is indicated by the flared edges (connections) between nodes. (B) Validation of modifier mechanisms across independent mutations result in an “inside-out” module, with the shared modifier (green circle) acting as a source hub on several conditioning or test mutations. An incipient network around *Nxf1^Mvb1^*, based on shared genetic mechanism, is illustrated. *Atrn^mgL^* and several intronic ETn-induced mutations are not affected by *Nxf1^Mvb1^* variation (gray). *Pitpna^vb^* and *Eya1^BOR^* have additional known modifiers (light green), adding modules to the incipient network.

Using epigenetically sensitive *Agouti* alleles and green fluorescent protein (GFP) transgenes that show variegated expression as phenotype reporters, Whitelaw and coworkers have recovered a substantial collection of ENU-induced *Modifier of murine metastable epialleles* dominant (*MommeD*) mutations. This approach creates an “outside-in” interaction network module, with multiple edges (interactions) converging on a single node (conditioning mutation or reporter gene) (Figure 2A). At least five of these mutations have been molecularly identified, encoding core components of chromatin regulatory complexes and a DNA methyltransferase [39]–[42]. The first recessive mutation identified from this screen, *MommeR1*, is an amino acid replacement allele of transcription factor *Foxo3a* [43]. Each of these mutations has independent phenotypes in addition to the modifier activity, including recessive lethality for the dominant alleles and female infertility for *Foxo3a*.

Another instructive example comes from an ENU screen for dominant enhancers of dominant white spotting in a *Sox10^lacZ^* ^/+^ model of Waardenburg syndrome [44]. Among 230 pedigrees, Pavan and colleagues recovered three transmissible modifiers of spotting (*Mos1,2,3*). Remarkably, these three induced mutations mapped to locations distinct from several previously identified modifiers of *Sox10* and from each other, suggesting that *Sox10* phenotypes are sensitive to perturbations at many positions in an extended genetic network. A collateral phenotype (cephalosyndactyly) and map position strongly suggested *Gli3* as the *Mos1* gene, which sequence and complementation analysis rapidly confirmed. *Mos2* was subsequently identified as a null allele of the RNA regulator *Magoh*, with severe haploinsufficiency phenotypes in brain development [45].

As illustrated in these examples, chemical mutagenesis allows efficient exploration of genetic networks unconstrained by prior hypothesis. Networks ascertained by this approach, in both examples, comprise modifier alleles that typically have independent deleterious phenotypes. Whether this is more often true of induced mutations or is a property of the specific networks tested, and whether milder alleles at the same genes might retain modifier effects without collateral phenotypes remains to be seen. Alternatively, networks based on variants in natural populations—or nominally wild-type strains—might highlight different genes and network connections, depending on biological trade-offs between modifier effect and collateral phenotypes in any gene's potential allele spectrum and the forces of selection through which a given variant has passed prior to discovery.

## Natural Variants: Keys to Genetic Plasticity?

While inbred mouse strains are often referred to as nominally “wild type,” each represents a different sampling of wild (and a few laboratory-derived) alleles across the genome. Inbred strains in aggregate represent an abundant source of captive variation [46], [47] and the vast majority of underlying genetic variants predate laboratory domestication [48]–[50]. This argues that the captive variants—on average—were sufficiently neutral to be sufficiently frequent among wild mice to be incorporated in the common laboratory strains, although some specific instances will be maladaptive by themselves or in combination with other variants. Modifier alleles among the nearly neutral majority of strain variants might be expected to favor, in comparison to mutagenesis experiments, either milder alleles or nodes in the relevant genetic networks with greater intrinsic plasticity—functional variation compatible with normal phenotype. Identification of network nodes with greater genetic plasticity may be especially useful for pointing out experimental and therapeutic approaches with higher likelihood to minimize collateral damage in modifying a specific condition. Three examples of early successes indicate some of the areas to which natural variants might contribute unique findings.

Studies in several laboratories have identified modifiers that alter *Apc^Min^*-dependent tumor phenotypes in a genetic cancer model [51]–[58]. *Modifier of Min-1* (*Mom1*) is the most well studied, beginning with its discovery by linkage analysis for intestinal tumor number nearly two decades ago [57]. Based on rough map location and strain distribution, the secreted phospholipase *Pla2g2a* was proposed as a positional and functionally variant candidate gene, with an apparent null allele caused by a single base insertion in *Apc^Min^* sensitive strains [59]. *Mom1* is semidominant and analysis of tumor DNA suggested that its effects are not cell autonomous, consistent with its identification as a secreted enzyme. Complementation studies confirmed that *Pla2g2a* accounted for a large fraction of the variance in tumor number [60] (an additional component, *Mom6*, was inferred to account for the full effect of the initial linkage [56], adding as a grace note that linkage peaks may be driven both by variants of large effect and by regions containing more than one contributing variant). Elevated expression of human *PLA2G2A* was subsequently associated with survival in gastric cancer patients [61], providing another example of cross-species validation. *Mom1* alleles in mice are widely distributed across inbred strains, suggesting an early origin. Re-sequencing [49], [62] and dense genotyping [48] data provide a partial answer. Review of published data shows that the *Mom1* single base insertion is a derived allele that arose on a unique haplotype and that at least 2 Mb of this interval is shared by all characterized *Mom1^S^*-allele strains. (Surprisingly, C57BL/6 and wild-derived MOLF/Ei strains share haplotype, 3630/3635 called variants, across 2.4 Mb around *Pla2g2a*, suggesting contamination of the MOLF/Ei stock with laboratory strains for this interval [63].) While the origin of this *Pla2g2a* allele is not yet clear, the circumstantial evidence warrants further investigation as a possible wild variant.

The *Heart failure modifier 2*, one of several mapped *Hrtfm* loci that modify the course of cardiomyopathy caused by overexpression of calsequestrin [64], was recently identified as an allele of the troponin-interacting kinase gene, *Tnni3k*, by a combination of fine mapping, strain distribution, and transgenic studies [65]. Molecular studies in a cell culture model implicated a single intronic SNP as the functional variant, creating an alternate 5′ splice donor site that results in a shifted open reading frame in ∼70% of *Tnni3k* transcripts in calsequestrin-resistant strains. To illustrate potential origin of this strain variant, we tabulated both functionally tested alleles [65] and correlated sequence data from additional strains (Table 1) [62]. This shows a broad distribution of each allele across accepted strain genealogies [66] for a region of modest haplotype diversity, suggesting an origin prior to development of laboratory strains and possibly wild-derived. Interestingly, subsequent work implicates the same *Tnni3k* variant in susceptibility to coxsackievirus-induced myocarditis [67]. If this variant is confirmed as a wild allele, it will be interesting to determine what factors might account for its accumulation despite the increased risk to cardiac challenges.

**Table 1:** TNNI3 interacting kinase–Heart failure modifier 2 (*Tnni3k^Hrtfm2^*) variant is widely distributed in laboratory mice.

Variant	Granby Farm-Derived^a^	Swiss Mice	China and Japan Stocks	Other Inbred	Wild Derived
***Casq1-*** **resistant variant (derived) and haplotype**	A/J, BALB/c, C3H, DBA, NZO	NOD, NZO			
**Share haplotype, variant not tested**	I/Ln, MA/My, SEA		DDK, KK	BUB, RIIIS	
**Share haplotype w/o resistant variant**					WSB (North America)
***Casq1-s*** **ensitive variant (ancestral)**	129P2, 129S1, AKR, C57BL/6, CBA	FVB			CAST (Asia), PWK (Europe)

a Strain categories are taken from [66]; Granby Farm-derived mice include both strains descended from Castle's mice and C57-related strains, both of which derived in part from A. Lathrop's stocks at Granby Farm. Strain-specific sequences from [62]. Inferred haplotypes from [48].

The *Modifier of vibrator 1* was originally detected as a semi-dominant modifier of neurodegeneration and premature death in *vibrator* mutant animals. The *Pitpna^vibrator^* mutation is an IAP-family endogenous retrovirus insertion into an intron of a phosphatidylinositol transfer protein gene, which reduces normal gene expression by competing with splicing of the adjacent exons [68]. Suppression by the CAST/Ei modifier allele occurs by elevating the level and proportion of normally spliced mRNA from the mutant gene. Positional complementation cloning identified the modifier as an amino acid substitution allele of mRNA nuclear export factor *Nxf1*, suggesting a previously unexpected role for canonical mRNA export machinery in splicing fidelity in at least some contexts [69]. Importantly, this dose-responsive effect on alternative splicing has been demonstrated for six out of seven mutations caused by sense-strand IAP insertions in host gene introns, but none of 15 mutations with other classes of insertional events [70]. This creates an “inside-out” network module, with a modifier node acting on several different mutations, some of which have additional known modifiers (Figure 2B). Of special interest, the suppressing *Nxf1* allele was found in a majority of wild *castaneus* isolates, confirming it as a natural variant, and haplotype analysis suggested directional selection, with a non-conservative amino acid replacement in *Nxf1* as the youngest variant on the most common wild haplotype [69].

Among modifiers not yet identified molecularly, the *Modifier of dactylaplasia* (*Mdac*) may impact several themes raised above, including selection, specificity, and epigenetic regulation. The functionally tested alleles are both well dispersed across inbred strains [71], [72], suggesting an early—and possibly wild—origin. The modifier acts on two different *Dac* alleles of *Fbxw4*, caused by independent insertions of MusD-family retrovirus elements at different sites [73], but has no obvious independent phenotype in reports from several different laboratories. Most interestingly, *Mdac* alleles alter both DNA methylation and the accumulation of inhibitory histone modifications on the *Dac* MusD elements [74], raising the intriguing possibility that this locus might act directly in epigenetic mechanisms, without strong collateral phenotypes. This would be interesting both as a phenotypically quiet epigenetic modifier and potentially as a tool for titrating allele strength of other MusD/ETn-induced mutations with similar epigenetic profiles.

Identifying modifiers on the basis of natural variants may require more resources than de novo mutations (once generated), but have the added value of generally highlighting alleles, genes, or networks whose manipulation appears well tolerated by the organism and largely compatible with normal function, having been vetted by selective pressures. In the ultimate goal of finding pressure points through which to modulate disease networks, this may prove advantageous.

## Going Forward: Tools for Accessing Strain Variation and Modifier Genes

How readily can we use modifier genes to predict sensitive pathways and nodes for therapeutic interventions? New tools and resources in mouse genetics should help to unclog the pipeline of modifier genes that have been detected but not molecularly defined. Maps of copy number variation [75] and comprehensive genome sequences of the most commonly used strains [62], [76] will be enormously powerful. The combination of predictive sequence variants plus expression data from increasingly facile array and RNA-Seq methods should allow comprehensive consideration of candidate variants across even broadly defined loci. Because modifier genes require the presence of the conditioning mutation, strain resources built for genome-wide shuffling of alleles [77] may have less value for this application (in requiring many crosses to access the variation), while others such as chromosome substitution panels [78], [79] may be useful for isolating individual modifier effects by limiting the need for de novo congenic strain construction. New tools for functional validation at fine scale are also essential to opening the pipeline of modifier gene studies in mice. Recent developments on this front include an increased diversity of strain-specific large-insert BAC libraries (http://bacpac.chori.org/, among others) for transgenic studies, larger-scale tools for engineering ES cells [80]–[82], and most recently germline-potent ES cells from previously refractory strains [83]. These resources, some anticipated a decade ago [84], [85] and some less expected, are only now widely available. This combination of tools and continuing innovations promises deeper insight into mammalian genetic architectures in health and disease, through the lens of modifier genes and mouse genetic diversity.

## Supporting Information

Table S1 A curated list of mouse modifier genes. The attached spreadsheet collects several examples of modifiers and a few exemplar interactions that may strain the strict definition of the term. Entries were manually curated from entries in the Mouse Genome Database [86], [87] with modifier, suppressor, or enhancer annotations; independent nested searches of PubMed for similar terms; authors' recollections; referrals from colleagues and suggestions from anonymous reviewers. We acknowledge that the list is incomplete and apologize to colleagues whose work we have missed by this limited search strategy. We have not independently reviewed the strength of linkage evidence for named loci. Molecularly identified modifier genes are emphasized with red text.
(XLSX)
